# Imitative Learning as a Connector of Collective Brains

**DOI:** 10.1371/journal.pone.0110517

**Published:** 2014-10-16

**Authors:** José F. Fontanari

**Affiliations:** Instituto de Física de São Carlos, Universidade de São Paulo, São Carlos, Brazil; Peking University, China

## Abstract

The notion that cooperation can aid a group of agents to solve problems more efficiently than if those agents worked in isolation is prevalent in computer science and business circles. Here we consider a primordial form of cooperation – imitative learning – that allows an effective exchange of information between agents, which are viewed as the processing units of a social intelligence system or collective brain. In particular, we use agent-based simulations to study the performance of a group of agents in solving a cryptarithmetic problem. An agent can either perform local random moves to explore the solution space of the problem or imitate a model agent – the best performing agent in its influence network. There is a trade-off between the number of agents 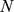 and the imitation probability 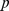, and for the optimal balance between these parameters we observe a thirtyfold diminution in the computational cost to find the solution of the cryptarithmetic problem as compared with the independent search. If those parameters are chosen far from the optimal setting, however, then imitative learning can impair greatly the performance of the group.

## Introduction

Imitative learning or, more generally, social learning offers a means whereby information can be transferred between biological or artificial agents, thus being a crucial factor for the emergence of social intelligence or collective brains [1]. Its relevance in this context is neatly expressed by Bloom: “Imitative learning acts like a synapse, allowing information to leap the gap from one creature to another” [2]. Not surprisingly, the advantages of this learning strategy were perceived and exploited by nature well before the advent of the human species as attested by its widespread use in the animal kingdom [3]–[6]. Regarding human behavior, we note that imitation as a mechanism of social learning was extensively studied by Bandura in the 1960s [7], [8] and that the sociocognitive approach to mental processing holds that all mental activity involves either representations of other people or the use of artifacts that have a social history [9], [10].

Social learning has inspired the design of several optimization techniques, such as the particle swarm optimization algorithm [11], [12] and the adaptive culture heuristic [13], [14]. Despite the success of these heuristics in producing optimal or near optimal solutions to combinatorial optimization problems, we know little about the factors that make cooperation effective, as well as about the universal character (if any) of the quantitative improvements that results from it [15]. The reason is probably that those heuristics and the problems they are set to solve are too complex to yield to a first-principle analysis. In this contribution we address these issues by tackling a simple combinatorial problem and by endowing the agents with straightforward search strategies in which the strength of collaboration is controlled by a single parameter of the model.

The combinatorial problem we consider here is a cryptarithmetic puzzle, i.e., a code in which the digits of the integer numbers in a sum are replaced by letters of the alphabet [16], [17]. The challenge is to find an assignment between letters and digits that satisfies the constraints of arithmetics as well as the condition that two different letters cannot be assigned to the same digit. In this sense, cryptarithmetic puzzles are typical of constraint satisfaction problems which play a central role in our understanding of human and computer problem solving competencies [18], [19].

We solve the cryptarithmetic problem using a group of 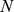 agents which, in addition to the capacity to carry out random local searches, can learn from (or imitate) a model agent – the best performing agent in their influence networks at a given trial. The influence network of each agent is obtained by picking 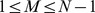 agents at random and without replacement from the 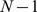 remaining agents in the group. The fully connected system corresponds to the case 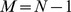. The frequency of the imitative or cooperative behavior is determined by the imitation probability parameter 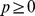. Hence our model exhibits two critical ingredients of a collective brain, namely, imitative learning and a dynamic hierarchy among the agents [2].

Our agent-based model conforms to the particle swarm paradigm in that the agents show a tendency to move towards the low cost regions of the solution space which were visited by members of their influence networks [11]. This tendency is a result of the imitation procedure that occurs with probability 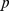. However, we relax the requirement that the agents are more likely to change if the move leads them to a region of lower cost, the so-called Law of Effect [10]. In particular, we allow the agents to move randomly in the solution space with probability 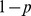 and it is this procedure that guarantees that, eventually, one agent will hit the solution of the cryptarithmetic problem [15]. We stress that, whereas the particle swarm algorithm or the adaptive culture heuristic may fail to find the solution of the puzzle because the search can get stuck in a local minimum of the cost landscape, our search procedure, which combines imitation and random changes, always finds the solution. Of course, the issue is how long it takes to do so.

The main performance indicator for the cooperative system is the total number of agent updates necessary to find the solution of the cryptarithmetic problem, which we define as the computational cost of the search. The baseline performance corresponds to the case 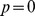 where the 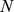 agents explore the solution space independently, resulting in a computational cost that does not depend on the value of 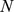, provided that this value is not too large compared to the size of the solution space. We find that, for a fixed value of the imitation probability 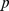, increasing the number of agents 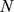 beyond a certain value impairs the group operation which can then perform much worse than in the case of the independent search. The following of a bad model is the culprit for the poor performance in this case. This harmful effect can be mitigated somewhat by reducing the size 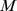 of the influence networks, so as to limit the influence of a model agent to only a fraction of the group. Most significantly, this finding implies that, for fixed 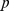 and 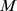, there is a value of group size 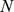 that minimizes the computational cost of the search. For instance, in such an optimal setting, say a fully connected system of 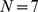 agents (hence 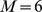) with imitation probability 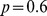, we find a thirtyfold decrease of the mean computational cost as compared with the baseline cost.

## Methods

First we will describe the particular cryptarithmetic problem the agents must solve, explain how the digit-to-letter identifications are encoded in strings and introduce the cost value associated to those strings. We will present also the elementary move that transforms any valid string into an adjacent valid string and so allows the full exploration of the solution space. Once these basic elements are introduced we will describe the mechanism of imitation between agents, thus completing the specification of the agent-based model we use to evaluate the efficacy of imitative learning in solving a complex task.

### The cryptarithmetic problem

Cryptarithmetic problems such as
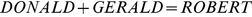
(1)are constraint satisfaction problems in which the task is to find unique digit assignments to each of the letters so that the numbers represented by the words add up correctly [16]. In the cryptarithmetic problem (1), there are 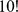 different digit-to-letter assignments, of which only one is the solution to the problem, namely, 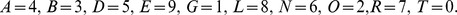 In fact, with this assignment the cryptarithmetic problem (1) is rewritten as the sum 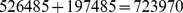 which accords with the arithmetic rules. We note that any other one-to-one correspondence between the 10 letters that appear in (1) and the 10 digits would violate those rules. This type of cryptarithmetic problem, in which the letters form meaningful words, are also termed alphametics [17] and were popularized in the 1930s by the *Sphinx*, a Belgian journal of recreational mathematics [16]. Of course, from the perspective of evaluating the performance of search heuristics on solving cryptarithmetic problems, the meaningfulness of the words is inconsequential, but in this contribution we will focus mainly on the alphametic problem (1). Nonetheless, we will offer evidence to support the validity of our conclusions by considering a few randomly generated cryptarithmetic problems as well.

A non-random search heuristics to solve cryptarithmetic problems requires the introduction of some arbitrary quality measure or cost value to each possible digit-to-letter assignment. For the alphametic problem (1) we encode a digit-to-letter assignment by the string 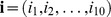 where 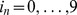 represent the 10 digits and the subscripts 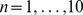 label the letters according to the convention
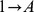


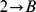


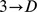


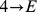


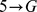


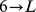


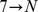


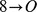


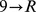


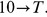
(2)


For example, the string 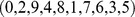 corresponds the the digit-to-letter assignment 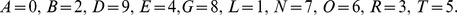 A somewhat natural way to associate a cost to a string 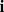 is through the expression [20]

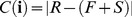
(3)where 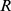 is the result of the operation (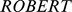), 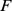 is the first operand (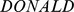) and 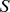 is the second operand (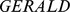). In our example we have 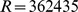, 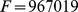 and 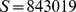 so that the cost associated to string 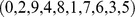 is 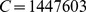. If the cost of a string is 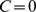 then the digit-to-letter assignment coded by that string is the solution of the cryptarithmetic problem. We must note that the cost value defined in eq. (3) applies to all strings except those for which 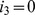 corresponding to the assignment 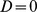, 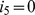 corresponding to the assignment 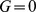 and 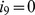 corresponding to the assignment 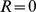. In principle, those are invalid strings because they violate the rule of the cryptarithmetic puzzles that an integer should not have the digit 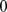 at its leftmost position. For those strings we assign an arbitrary large cost value, namely, 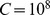, so that they can be considered valid strings and hence part of the solution space.

In addition to the assignment of the cost values to all 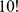 strings that code the possible digit-to-letter mappings for the alphametic problem (1), we introduce also an elementary move that connects two valid digit-to-letter mappings. We define the elementary move as follows. Starting from a particular digit-to-letter mapping, say 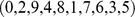, we choose two letter labels at random and then interchange the digits assigned to them. For example, say we pick letter labels 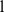 and 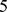, then the mapping that results from the application of the elementary move is 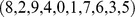. Clearly, the repeated application of our elementary move is capable of producing all 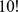 strings starting from any valid digit-to-letter mapping.

### Imitative learning

The system is composed of 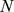 agents or strings which represent valid digit-to-letter assignments as described before. Each agent is connected unidirectionally to exactly 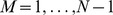 distinct, randomly chosen agents in the system. We will refer to those agents as the ‘influencers’ of the target agent. More specifically, for each agent we sample 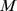 influencers from the 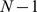 remaining agents without replacement. The extreme case 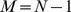 corresponds to the fully connected network. An agent has a probability 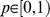 of copying a digit-to-letter assignment from a model string in its group of influencers, and probability 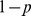 of performing the elementary move. We choose the model string as the lowest cost string among the 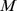 influencers of the target agent. If the cost associated to the target string is lower than the cost of the model string then the copying process is aborted.

To illustrate the copying process let us assume for the sake of concreteness that the target agent is our already familiar example string 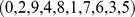, whose cost is 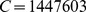, and that the model string is 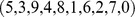 whose cost is 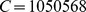. In the copying process the target agent selects at random one of the distinct digit-to-letter assignments in the model string and assimilates it. In our example, the distinct assignments occur at the letter labels 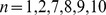. Say that the letter label 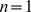, which corresponds to the assignment 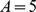 according to our convention (2), is chosen. To assimilate this assignment the target agent needs to reassign the digit 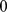 to the letter label which was previously assigned to digit 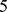 so that the resulting string becomes 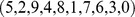, whose cost is 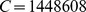. As expected, a result of the imitative learning process is the increase of the similarity between the target and the model strings. The case 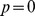 corresponds to the baseline limit where the 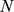 agents perform independent searches. The specific copying procedure proposed here was inspired by the mechanism used to model the influence of an external media [21]–[23] in Axelrod's model of culture dissemination [24]. It is important to note that in the case the target string is identical to the model string, as well as in the case the cost of the target string is lower than the cost of the model string, the opportunity of update is wasted.

We may interpret the imitation (or copying) process of a model string as a blackboard cooperation system where a central control exhibits hints (i.e., the lowest cost string) in a public space [15], [25], but here we prefer to use the interpretation of learning by imitation in a social context. Nevertheless, since the process of imitation results in an effective collaboration among agents, in the sense that there is an exchange of information between them, we refer to this search strategy as collaborative search to contrast with the independent search which occurs when the copying process is turned off, i.e, the imitation probability 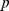 is set to zero.

### Search dynamics

We begin by generating the 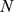 influence networks, i.e., a group of 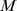 influencers for each agent. These networks are kept fixed during the entire search. In this initial stage, at trial number 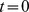, we also associate a random digit-to-letter assignment (a valid string) to each agent and determine its corresponding model string by evaluating and comparing the cost values of its 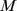 influencers.

A new trial begins with the choice of the update order of the 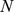 agents, so that at the end of the trial all 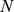 agents are updated. The agent to be updated – the target agent – has the possibility to imitate its model string or perform the elementary move with probabilities 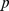 and 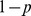, respectively. After update, we must re-evaluate the model string status in all groups of influencers to which the target agent belongs. After all 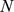 agents are updated we increment the trial number 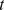 by one unit and check whether any string has cost zero, in which case the search is halted. The trial number at which the search ends or, alternatively, the number of trial to success is denoted by 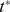.

Except for the independent search (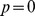), the update of the 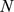 agents is not strictly a parallel process since the model strings may change several times within a given trial. Nonetheless, since in a single trial all agents are updated, the total number of agent updates at trial 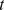 is given by the product 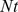.

## Results

The efficiency of a search strategy is measured by the total number of agent updates necessary to find the solution of the cryptarithmetic problem (i.e., 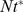) and in the following we will refer to this measure as the computational cost of the search. Since we expect that the typical number of trials to success 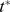 scales with the size of the solution space (i.e., 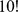), we will present the results in terms of the rescaled variable 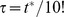. For the purpose of comparison we will consider first the independent search strategy where the agents can perform the elementary move only (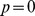) and then the general cooperative search (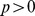) where the agents are also allowed to imitate their models.

### Independent search

**Figure 1:**
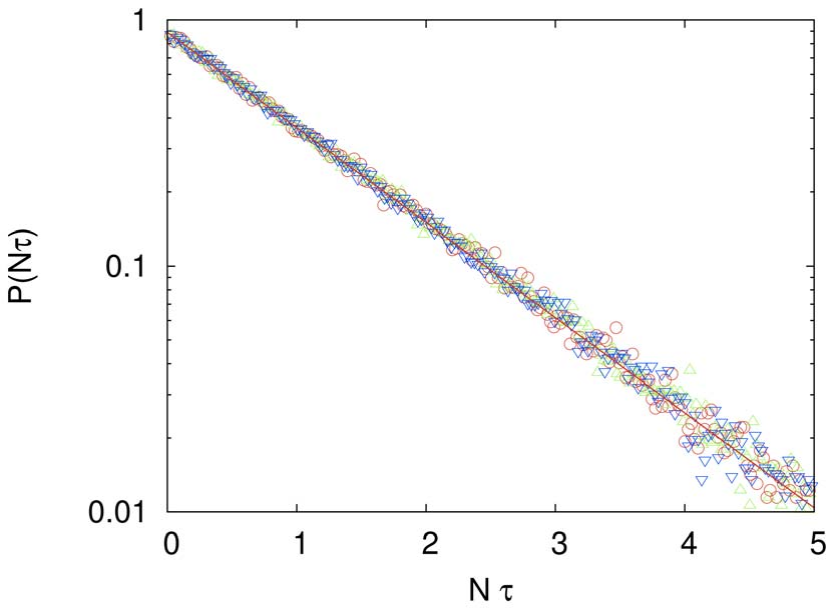
Exponential distribution of the rescaled computational cost for the independent search. Probability distribution 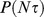 that a search employing 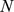 independent agents finds the solution of the cryptarithmetic problem (1) using a total of 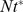 updates for 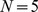 (green triangles), 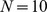 (blue inverted triangles) and 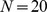 (red circles). Here 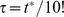 is the ratio between number of trials to success and the size of the solution space. These distributions were generated using 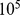 independent searches for each 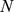. The solid straight line is the exponential distribution 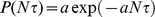 with 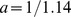. The influence network size 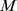 does not affect these results since imitation is not allowed in this case.

In this case there is no imitation and so the influence networks have no role in the outcome of the search. The main results of the independent search are summarized in Figure 1, which shows the probability distribution 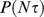 of the rescaled computational cost 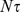 of the search for several system sizes. The data is very well fitted by the exponential distribution 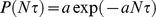 with 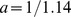 which is shown by the solid straight line in the figure.

As expected, the mean rescaled computational cost 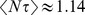 is insensitive to the system size provided that 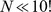, but the finding that it does not equal 1 is somewhat surprising. In fact, if we replace our elementary move by a global move in which the entire string is generated randomly at each update then we find that this mean equals 1, as expected. The reason that our elementary move is slightly less efficient than the global move in exploring the solution space is because it is not too unlikely to reverse a change made by the elementary move. For example, the probability to reverse a change in a subsequent trial is 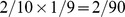 for the the elementary move, whereas it is 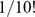 for the global move.

### Cooperative search

As pointed out before, the cooperation among agents stems from the possibility that they copy potentially relevant digit-to-letter assignments from the model strings in their influence networks. We will consider first the fully connected system where 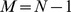 and then the partially connected systems where 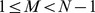.

#### Fully connected system

**Figure 2:**
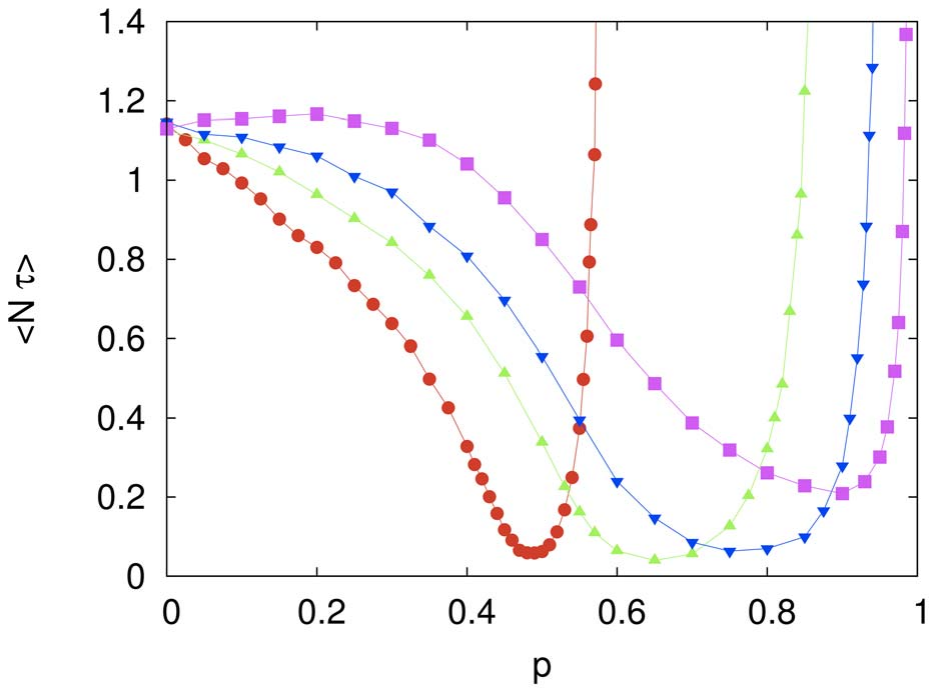
The effect of the imitation probability on the computational cost of the fully connected system. The symbols represent the mean rescaled computational cost 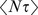 for cooperative systems of size 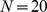 (red circles), 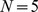 (green triangles), 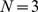 (blue inverted triangles) and 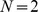 (magenta squares). The independent variable 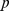 is the probability that an agent will copy a digit-to-letter assignment from the model string, chosen as the lowest cost string in the entire system. The influence network size is 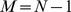. Each symbol represents an average over 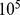 searches and the lines are guides to the eye. The error bars are smaller than the size of the symbols.

Figure 2 shows how the mean rescaled computational cost is affected by varying the imitation probability 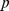 while the number of agents 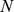 is kept at a fixed value. For 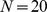 and 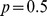 we observe a twentyfold decrease of the mean cost in comparison with the cost of the independent search, which corresponds to 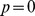 and yields 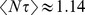. This is a remarkable evidence of the power of imitative learning to speed up the search on the solution space of the cryptarithmetic problem. In the limit 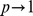 one expects the computational cost to diverge since the solution space cannot be fully explored as the option for the elementary move is never made in this limit. This harmful effect of learning by imitation becomes more pronounced as the number of agents increases.

**Figure 3:**
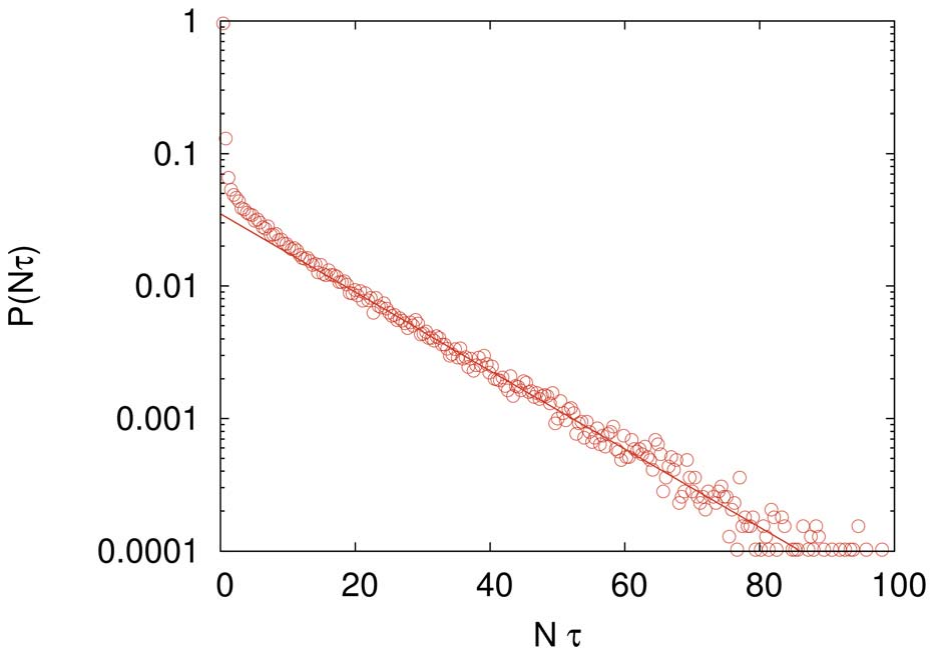
Deviation from the exponential distribution for a large imitation probability. Probability distribution 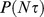 of the rescaled computational cost for a search employing 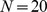 fully connected agents with imitation probability 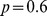. The mean of this distribution is 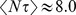. The solid straight line is the fitting function 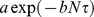 with 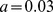 and 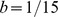 in the regime of large cost. The influence network size is 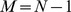. The distribution was generated using 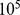 independent searches.

In the region where the mean computational cost decreases monotonically with increasing 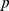 (e.g., 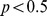 for 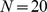) we found that the probability distribution of the computational cost is well described by an exponential distribution, in the sense that the ratio between the standard deviation and the mean is always very close to 1. (We recall that this ratio equals 1 for an exponential distribution.) However, in the region where 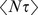 increases with increasing 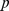 we found that in the low cost regime 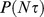 gives values significantly greater than those predicted by an exponential distribution, as illustrated in Figure 3, though those values are not greater than those obtained in the case of the independent search (see Figure 1).

**Figure 4:**
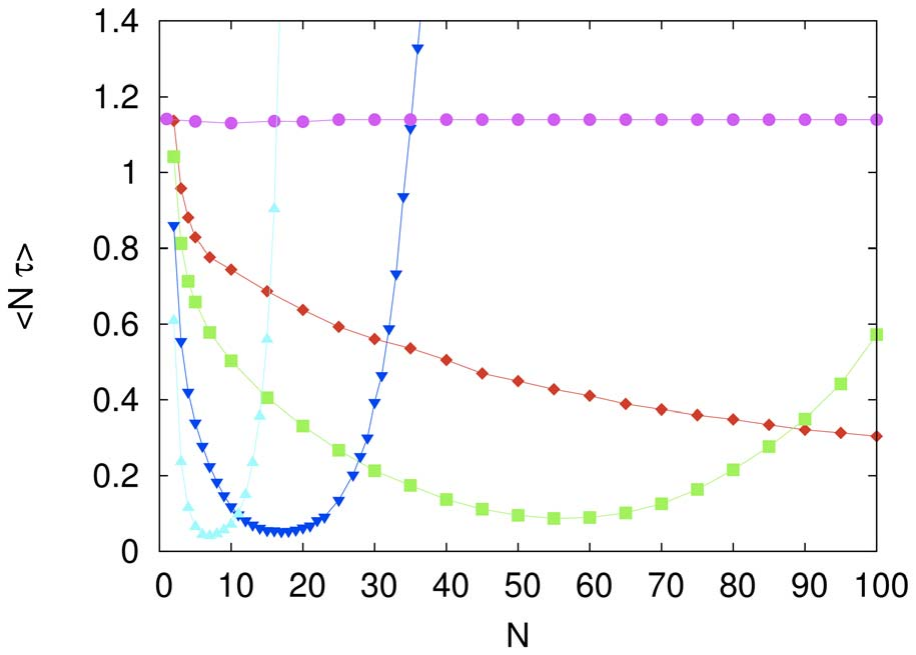
The effect of group size on the computational cost of the fully connected system. The symbols represent the mean rescaled computational cost 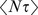 for the imitation probability 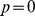 (magenta circles), 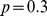 (red diamonds), 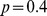 (green squares), 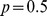 (blue inverted triangles) and 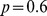 (cyan triangles). The independent variable 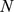 is the number of agents in the system. The influence network size is 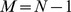. Each symbol represents an average over 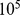 searches and the lines are guides to the eye. The error bars are smaller than the size of the symbols.

The effect of increasing the number of agents 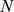 for a fixed value of the imitation probability 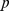 is summarized in Figure 4. The mean computational cost of the cooperative system exhibits a non-monotonic dependence on 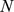, except in the case of the independent search (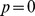) when it takes on a constant value. The benefit of cooperation is seen in this figure by the initial decrease of the computational cost as the number of agents increases. However, for all 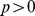 we find that the presence of too many agents greatly harms the performance of the system and that for a fixed 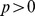 there exists an optimum value of 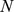 that maximizes the search efficiency of the cooperative system. For instance, although not shown in the scale of Figure 4, the minimum computational cost for 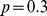 occurs at 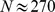. The efficiency at this optimum, however, is not affected significantly by the choice of the parameters 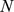 and 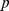. In other words, the costs corresponding to the minima shown in Figures 2 and 4 are not very sensitive to changes in 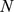 and 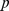, respectively. In particular, for the parameter settings we have explored, the best efficiency 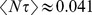 is achieved for 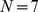 and 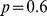 and amounts to a thirtyfold speed up with respect to the independent search.

We conjecture that the reason the efficiency of the cooperative system deteriorates as 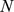 increases beyond its optimum value (e.g., in the range 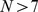 for 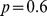 as shown in Figure 4) is that for 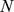 not too small there is a good chance that the cost of one of the strings is significantly lower than the cost of the other 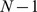 strings. Provided 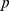 is not too small too, this string may remain as the model string for a few trials thus biasing the search to the vicinity of the model string. In the (typical) case that the model string is far from the solution of the cryptarithmetic problem, imitative learning may lead to the observed impairment of the performance of the cooperative system. In sum, the following of a bad leader is likely the culprit of the poor performance of the system.

To check the validity of this conjecture we calculate the mean number of consecutive trials for which a cost value stays as the lowest cost among the 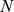 strings. The procedure to obtain this quantity, which we denote by 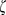, is straightforward. At trial 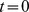 we evaluate the cost of the 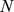 strings and record the minimal cost among them. Then at the next trial 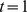, after the 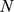 strings are updated, we re-evaluate again their costs and record the minimal cost. If the minimal cost at 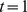 is different, i.e., greater or less, than the minimal cost at 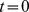 we say that a change event has occurred. The comparison of the values of the minimal costs at consecutive trials is repeated and the cumulative number of change events is recorded until the solution is found at 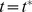. The desired quantity 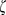 is given simply by the ratio between the total number of change events and the total number of trials 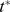. Hence for each search we obtain a single value for 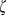, which can then be interpreted as the mean number of trials between consecutive change events or as the mean duration of the stases for that search.

**Figure 5:**
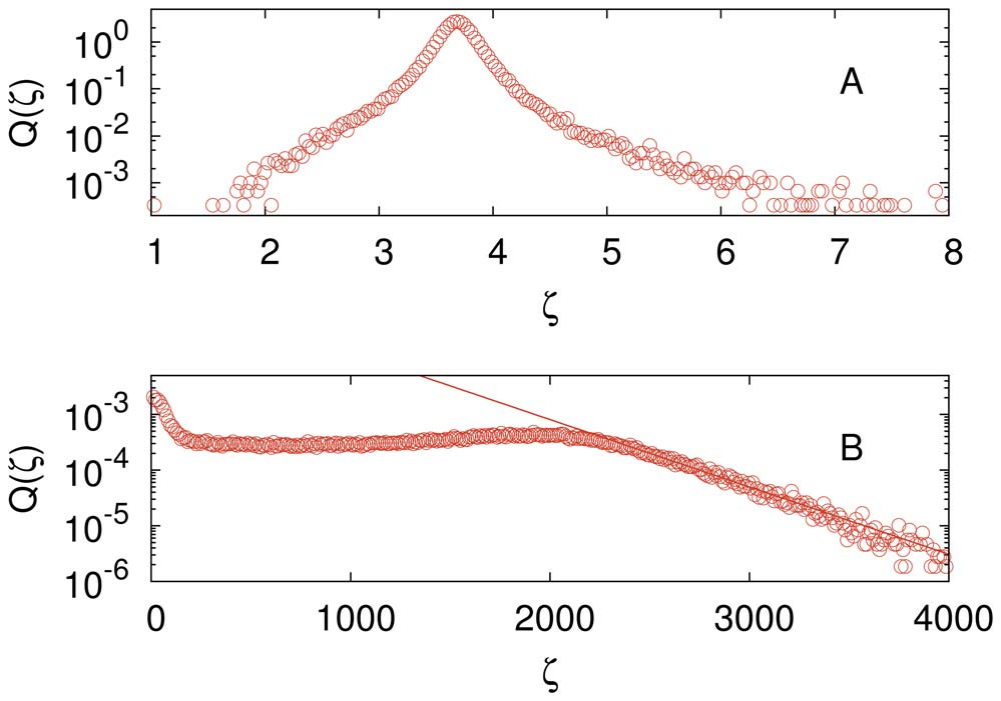
Probability distribution of the mean duration of the stases in a search. Probability distribution of the mean number of trials 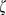 for which a cost value stays as the lowest cost among the 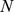 solutions in 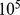 searches for the imitation probability 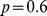 in a fully connected system. Panel **A**: 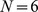 (low computational cost regime). Panel **B**: 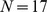 (high computational cost regime). The slope of the straight line shown in the semi-log scale of panel **B** is 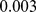. The influence network size is 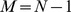.

In Figure 5 we present the probability distribution 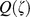 using 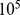 searches for the imitation probability 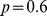 and two representative values of 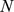. Figure 5**A** shows this distribution for 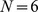, which corresponds to a regime of low computational cost according to Figure 4. We observe a pronounced maximum at 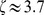 so that in most searches the model cost remains unaltered for 3 to 5 trials. This is an optimum scenario since no string stays on the top tier long enough to influence the entire system. For 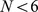, we find that 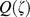 exhibits a similar shape but the maximum becomes sharper and its location is shifted towards lower values of 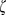 as 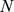 decreases. Figure 5**B**, which shows the results for 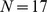, reveals a very different scenario: the distribution 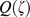 exhibits a plateau indicating that the model cost remains unchanged for hundreds to a few thousands trials. For very large values of 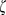, the distribution 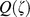 seems to exhibit an exponential decay to zero, namely, 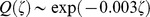. We stress that for the two cases exhibited in Figure 5 the probability that an agent will imitate the model rather than perform an elementary move is the same, namely 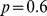, and so the qualitative differences reported in the figure are due solely to the change on the number of agents.

#### Partially connected system

**Figure 6:**
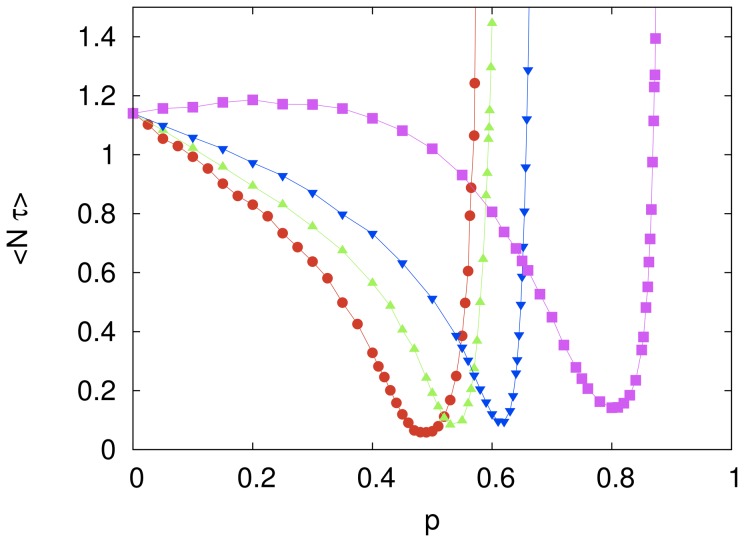
The effect of the imitation probability on the computational cost of partially connected systems. The symbols represent the mean rescaled computational cost 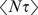 for a system composed of 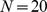 agents, each one connected to 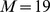 (red circles), 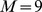 (green triangles), 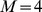 (blue inverted triangles) and 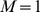 (magenta squares) influencers. The independent variable 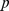 is the imitation probability. Each symbol represents an average over 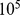 searches and the lines are guides to the eye. The error bars are smaller than the size of the symbols.

**Figure 7:**
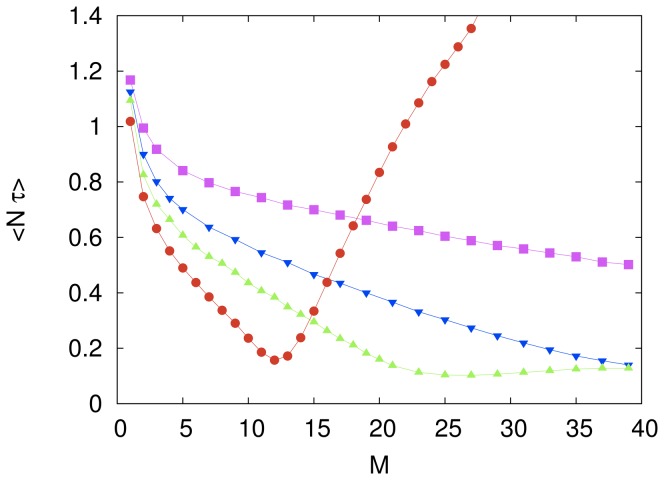
The effect of the number of influencers on the mean computational cost. The symbols represent the mean rescaled computational cost 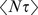 for a system composed of 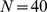 agents and imitation probability 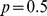 (red circles), 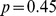 (green triangles), 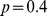 (blue inverted triangles) and 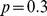 (magenta squares). The independent variable 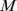 is the size of the group of influencers of each agent. Each symbol represents an average over 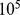 searches and the lines are guides to the eye. The error bars are smaller than the size of the symbols.

If the poor performance of large collaborative systems based on imitative learning is due to the influence of bad models then a natural way to reduce this harmful effect is to limit the influence of those models. This was the motivation to introduce the influence networks scheme where each agent picks its model among 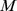 randomly chosen agents predetermined at the beginning of the search. In fact, Figure 6 shows that the reduction of the connectivity of the agents increases somewhat the range of values of the imitation probability 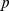 for which the cooperative system outperforms the system composed of independent agents. More pointedly, for 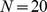 this range is extended from 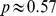 for 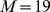 to 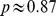 for 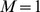. In addition, the value of the optimal mean computational cost does not seem to vary significantly with 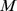. Figure 7 offers another perspective on the role of the number of influencers 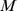. It shows that for small values of the imitation probability the fully connected system (i.e., 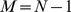) exhibits the best performance. However, as 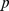 increases (e.g., 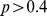 for 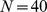), the optimal performance is obtained with partially connected systems. Moreover, we found that for any fixed value of 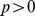 and 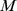 the performance of the system is always impaired when the number of agents 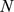 is very large. Finally, we note that similarly to our findings for the fully connected system, the probability distribution of the computational cost 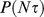 departs significantly from an exponential distribution only in the regions where the mean computational cost becomes an increasing function of the control parameters of the model.

#### Random cryptarithmetic problems

**Figure 8:**
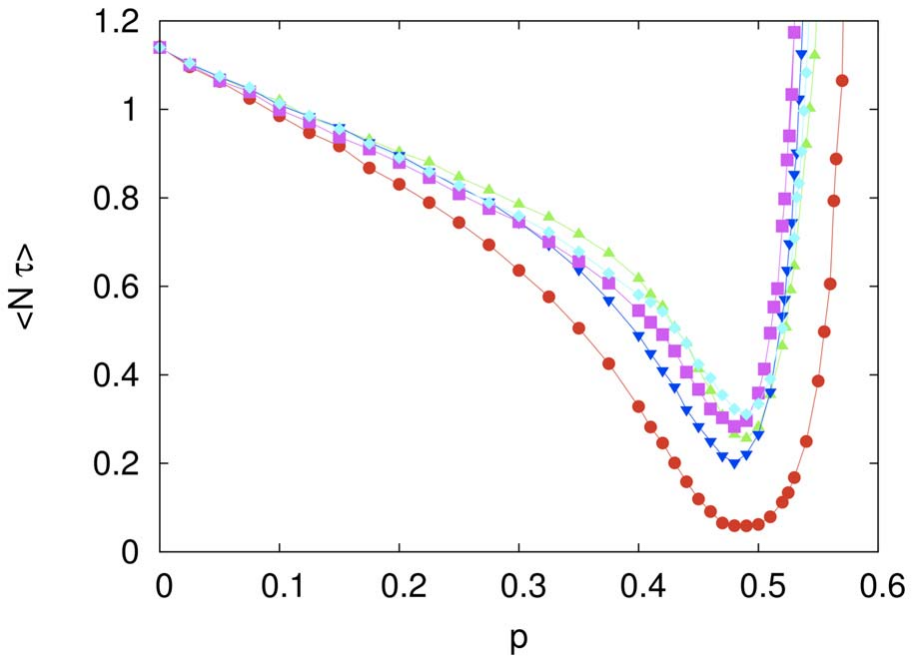
Computational cost of the alphametic problem and of four random cryptarithmetic problems. The mean rescaled computational cost for the alphametic problem 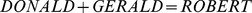 (red circles) and for four ten-letter random cryptarithmetic problems with a unique solution (blue inverted triangles, magenta squares, cyan diamonds and green triangles). The symbols represent the mean rescaled computational cost 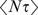 for a system composed of 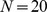 fully connected agents. The independent variable 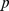 is the imitation probability. The influence network size is 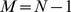. Each symbol represents an average over 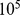 searches and the lines are guides to the eye. The error bars are smaller than the size of the symbols.

In order to verify the generality of our findings, which were obtained for the specific alphametic problem 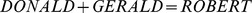, we have considered a variety of random cryptarithmetic problems with 10 letters and a unique solution, so that the sizes of their solution spaces are the same as that of the alphametic problem. The comparison between the mean computational costs to solve four such random problems and our alphametic problem is shown in Figure 8 for the fully connected system. The results are qualitatively the same, as expected. The alphametic problem, however, was somewhat easier to solve by the cooperative system than the random problems, perhaps because of the coincidence of the last three letters (“ALD”) in the first and second operands. Interestingly, the independent system (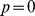) cannot distinguish between the problems but the cooperative system (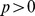) can, and this distinction is most pronounced when the parameters are set so as to achieve the optimal performance. It is as if the cooperative system had adapted to the specific task posed to it. We expect that our conclusions remain valid, in a qualitative sense of course, for any constraint satisfaction problem characterized by a rugged cost landscape.

## Discussion

Rather than offer any novel method to solve cryptarithmetic problems, our aim in this contribution is to assess quantitatively the potential of imitative learning as the underlying mechanism – the critical connector – of collective brains [2]. Here imitative learning is implemented by allowing an agent to copy clues from the best performing agent – the model agent – in its group of influencers. More pointedly, at trial 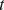 each agent has the probability 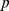 of imitating the model and the probability 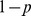 of executing a random rearrangement of the digit-to-letter mapping which is its guess to the solution of the cryptarithmetic problem. In an optimal setting, say a fully connected system of 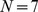 agents with imitation probability 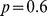, we find a thirtyfold decrease of the mean number of trials needed to find the solution of the problem (i.e., of the mean computational cost), as compared with the case 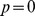 when the agents search the solution space independently (see Figure 4).

In the optimal setting, as well as in the regions where the computational cost is a decreasing function of the control parameters of the model, the probability distribution of the computational cost is given by an exponential distribution, rather than by a lognormal distribution as predicted by a general theory of cooperative processes [15], [26]. In fact, the reason the cooperative scheme implemented in [15] is so efficient is that all discovered digit-to-letter assignments that add up correctly modulo 10 for at least one column are permanently exposed as hints in a blackboard for use by all agents, which can pick a hint at each trial. There is no place for any kind of learning in that scenario since in the case there are no hints in the blackboard or the agent has already used the chosen one, the target agent selects at random a complete digit-to-letter mapping, which is totally uncorrelated to its previous mapping. The imitative learning interpretation of our cooperative scheme is only possible because in our case the elementary random move is local (i.e., solely two digit-to-letter assignments are changed in the entire mapping) and therefore preserves the identity of the target agent.

Most significantly, for fixed values of the imitation probability 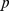 and of the number of influencers 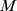, we find that increasing the number of agents 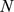 beyond a certain quantity impairs the working of the cooperative system, which then performs much worse than if the agents had executed independent searches. Our analysis indicates that the following of a bad model is the culprit of the poor performance of the system in this case. In that sense, the efficacy of imitative learning could be a factor determinant of group size [27]. In contrast to the cognitive load that constrains the number of individuals with whom it is possible to maintain stable relationships and leads to Dunbar's number for primates [28], the group size here (i.e., the value of 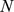 corresponding to the lowest computational cost) does not stem from a limitation of the neocortical processing capacity of the individuals. Rather, it is a property of the group of agents as a whole, since for any fixed non-vanishing value of the imitation probability, which may be seen as an individual trait, a too large number of agents, which is a group property, will impair the performance of the cooperative system. Of course, if 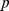 were allowed to decrease with increasing 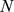 then the system could be maintained at the highest level of perform regardless of the group size (see Figures 2 and 4). In other words, in order to perform at the optimal level a system based on imitative learning should decrease the frequency of the interactions among individuals as its size increases.

To conclude, our findings indicate that imitative learning has a great potential to improve the task-solving capability of a group of agents, provided the model parameters – number of agents (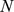), imitation probability (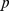) and number of influencers (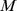) – are not too far from their optimal values. In the cases that 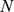 or 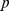 are too large, the imitative learning strategy leads the cooperative system astray, in a sort of maladaptive behavior that has actually been observed in fishes [29]. It would be interesting to find out what ingredients one should add to our model in order to prevent the catastrophic effect of imitative learning on large populations.
